# A Randomized Cross-Over Field Study of Pre-Hydration Strategies in Dogs Tracking in Hot Environments

**DOI:** 10.3389/fvets.2020.00292

**Published:** 2020-06-03

**Authors:** Greta M. Niedermeyer, Elizabeth Hare, Leslie K. Brunker, Richard A. Berk, Kathleen M. Kelsey, Tracy A. Darling, Jess L. Nord, Kasey K. Schmidt, Cynthia M. Otto

**Affiliations:** ^1^Department of Clinical Sciences and Advanced Medicine, School of Veterinary Medicine, University of Pennsylvania, Philadelphia, PA, United States; ^2^Penn Vet Working Dog Center, School of Veterinary Medicine, University of Pennsylvania, Philadelphia, PA, United States; ^3^Dog Genetics, LLC, Astoria, NY, United States; ^4^Department of Criminology and Statistics, University of Pennsylvania, Philadelphia, PA, United States; ^5^College of Veterinary Medicine, Washington State University, Pullman, WA, United States

**Keywords:** sports medicine, electrolytes, thermoregulation, working dogs, field study

## Abstract

The objective of this study was to evaluate 4 pre-exercise hydration strategies (oral water, chicken-flavored water, chicken-flavored oral electrolyte solution, and subcutaneous electrolyte solution) in working dogs conducting rigorous tracking operations in hot and arid conditions. In a randomized cross-over field study, 7 Border Patrol Search, Trauma, and Rescue (BORSTAR) Unit dogs working/training out of Fort Bliss in El Paso, Texas were randomly assigned to one of 4 different hydration strategy treatments each day for 4 days of study participation. Dogs were provided hydration treatment prior to running 2 separate one-mile tracks and were offered water while tracking. Body weight, blood, and urine were collected at the beginning of the study day and at the completion of each track. Core body temperatures were recorded using internal temperature sensing capsules. The impact of hydration strategy on change in weight, peak temperature, and serum chemical, hematological, and urinary parameters were analyzed using the COIN procedure in R^a^. Compared to the other 3 hydration strategies, dogs receiving chicken-flavored water had higher blood creatine kinase values at the end of the second track (*p* = 0.0361). Otherwise, hydration strategy had minimal effects on blood or urine parameters. Total fluid intake was lower with water only compared to the other three hydration strategies. Dogs developed elevated core body temperatures (median 41°C; 106°F) without signs of heat exhaustion or heat stroke. Alternate hydration strategies increased total fluid intake compared to water alone; however, chicken-flavored water resulted in increased markers of muscle injury suggesting electrolyte-enriched strategies may have an advantage as a hydration strategy. Additionally, electrolyte-enriched fluids before exercise may help these dogs maintain lower peak temperatures.

## Introduction

Military and other working dogs are critical for U.S. security and aid in border control as well as natural disaster response. Exercise-induced hyperthermia limits the ability of dogs to perform physically (1) and is one of the few preventable causes of death or euthanasia in MWD (2, 3). In a study analyzing reasons for discharge in MWD, heat stroke was the most common non-behavioral reason for dogs <5 years old to be discharged (4). Additionally, heat stress followed gunshot wounds and explosion/blast wounds as the third most common cause of death in MWD deployed in Iraq and Afghanistan (5).

The ability to thermoregulate and avert heat stress and its progression to heat stroke are influenced by work, ambient conditions, acclimatization, and hydration (3, 6–8). Since working dogs are often required to perform physically challenging tasks in adverse environmental conditions (e.g., high temperatures), efforts to mitigate the impact through improved acclimatization and hydration are beneficial.

Acclimatization or adaptation to environmental conditions allows dogs to better withstand hyperthermia, whether environmental or exercise-induced (8). A heat-acclimated animal (adapted under artificial conditions) is better able to tolerate longer exposures to heat as well as more extreme heat (9). Acclimation occurs as the animal adapts to better dissipate heat, produce less heat, and under certain conditions expand the safe range of core body temperatures (9). A heat-acclimated state results in lower core body temperature, reduced heart rate, elevated cardiovascular reserve, and increased evaporative cooling (9). Bruchim demonstrated that together with physical training, heat acclimatization resulted in a decreased rise in rectal temperature and heart rate in MWD following a physical performance test despite increased test intensity (10). While Bruchim showed dramatic changes, these results were measured after ~6 and 18 months of acclimatization (10). Partial acclimatization is thought to occur within 10–20 days, but full acclimatization can require up to 2 months (11).

Acclimatization is not always an achievable goal since working dogs are sometimes called to different climates with no time for acclimatization [e.g., When search and rescue (SAR) dogs from cooler climates were sent to Haiti following the 2010 earthquake (12)]. Additionally, hypohydration decreases heat tolerance regardless of acclimatization (13).

Hydration management provides a promising approach to reduce the risk of heat stress and heat stroke both in an acute setting when acclimatization is not possible and in concert with acclimatization. Yet, hydration management can pose a challenge in working dogs during demanding situations; dehydration was the most common health issue in dogs that responded to the earthquake in Haiti and was reported by handlers of SAR dogs that responded to 9/11 (12, 14).

Despite the importance of working dogs and the significance of heat stroke and dehydration, few studies have compared the safety and efficacy of various hydration strategies used in the field (15, 16). In a previous study comparing oral water, an oral electrolyte solution, and subcutaneous fluids, hydration strategy had only minor effects on physiological parameters and no detectable effect on behavioral parameters in vehicle-screening dogs working at the Sarita, Texas checkpoint, although dogs did increase their fluid consumption and hydration when offered a chicken-flavored oral electrolyte solution (16). It is unknown whether these same trends would hold up in more extreme conditions (i.e., high heat, no shade, and rigorous activity). To our knowledge, there are no studies evaluating hydration strategies in working dogs under these conditions or comparing the effects of a flavored oral electrolyte solution to flavored water.

The purpose of this study was to investigate the impact of four pre-exercise hydration strategies—oral water (W), chicken flavored water (CHK), chicken-flavored oral electrolyte solution (OES), and subcutaneous electrolyte solution (SCE)—on dogs tracking at the border in El Paso, Texas as part of the Border Patrol Search, Trauma, and Rescue (BORSTAR) unit. We hypothesized that hydration method would neither affect clinical parameters, including core temperature, in the dogs nor be associated with any adverse effects.

## Materials and Methods

### Ethics Statements

The protocols used in this study were reviewed and approved by both the University of Pennsylvania and US Army Medical Research and Materiel Command Institutional Animal Care and Use Committees (USAMRCM #SO120002, UPenn IACUC protocol #804293).

### Animals

Seven Border Patrol dogs working for the Customs and Border Protection BORSTAR Unit based out of Fort Bliss in El Paso, Texas were invited based on canine and handler availability during the experimental period. All dogs were trained to track and trail humans traversing the open desert. All dogs worked, trained, and were kenneled at the same facility, and all lived with their handlers. All dogs were deemed healthy and in good condition based on physical examination by a veterinarian (CMO) prior to starting the study. The age, breed, sex, neuter status, body condition, physical examination parameters, and medical and work history were collected from the handlers on all dogs. Exclusion criteria included any canine illness or injury, request of the handler not to participate, or any adverse events.

### Experimental Protocol

**Figure 1:**
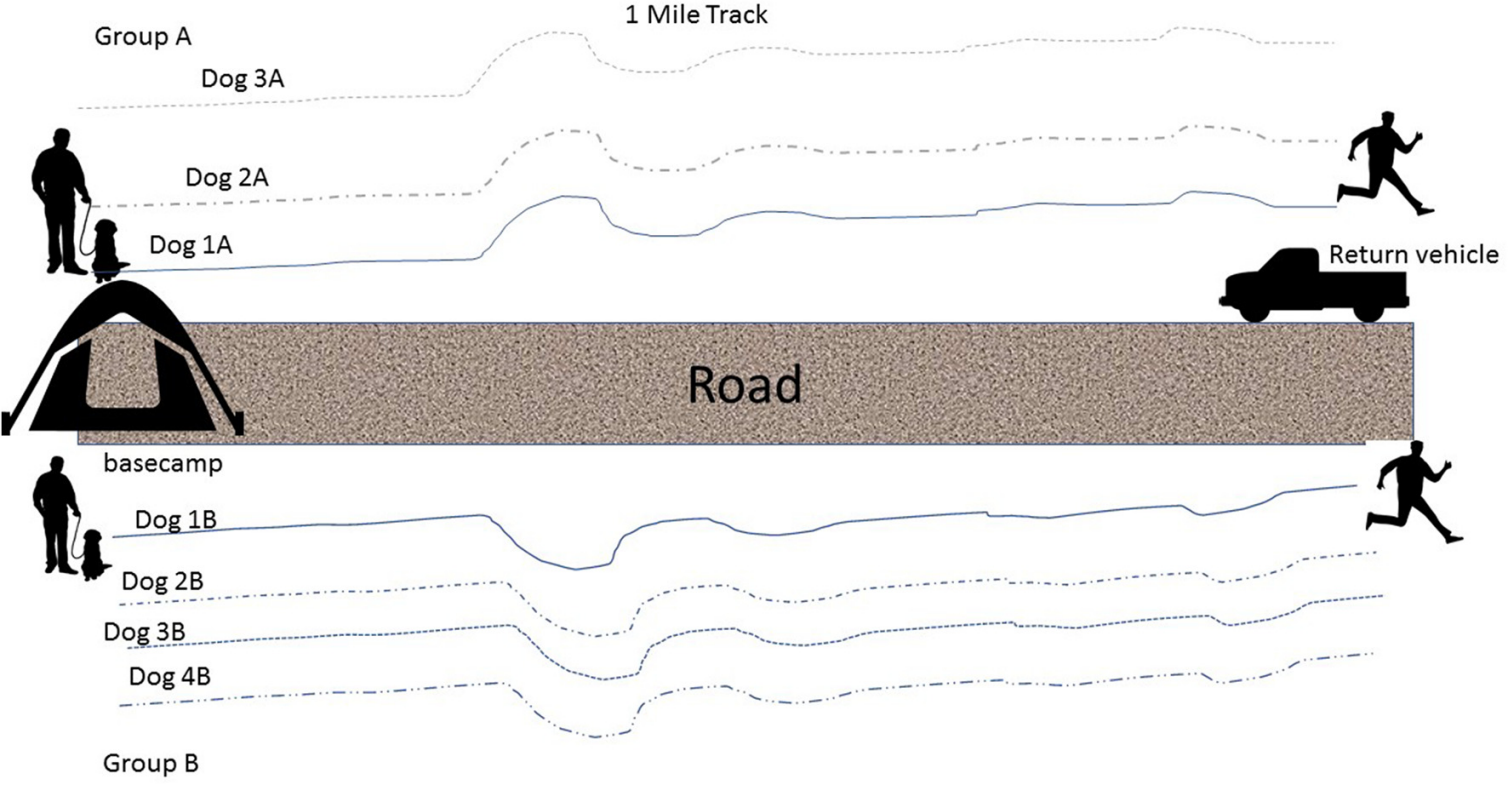
An illustration of the tracks that were laid for each dog during each of the tracking sessions (Track 1 and Track 2).

In this cross-over design, dogs were randomly assigned to each one of 4 treatment protocols over the 4 days of study participation. Study days were limited to alternating days to allow handlers time to attend mandatory training classes on base: Tuesday and Thursday during week 1, Monday and Wednesday during week 2. In a remote wilderness area, one-mile tracks were laid down by handlers or trainers ~30 min prior to the start of each dog's tracking session (each dog followed a fresh track). (Figure 1) Tracking order was randomly assigned and each dog completed a one-mile track (Track 1), rested, and then completed a second one-mile track (Track 2). Handlers took breaks and doused (i.e., wet down for cooling) their dogs with water during Track 1 and Track 2 as they would normally during a working session.

**Figure 2:**
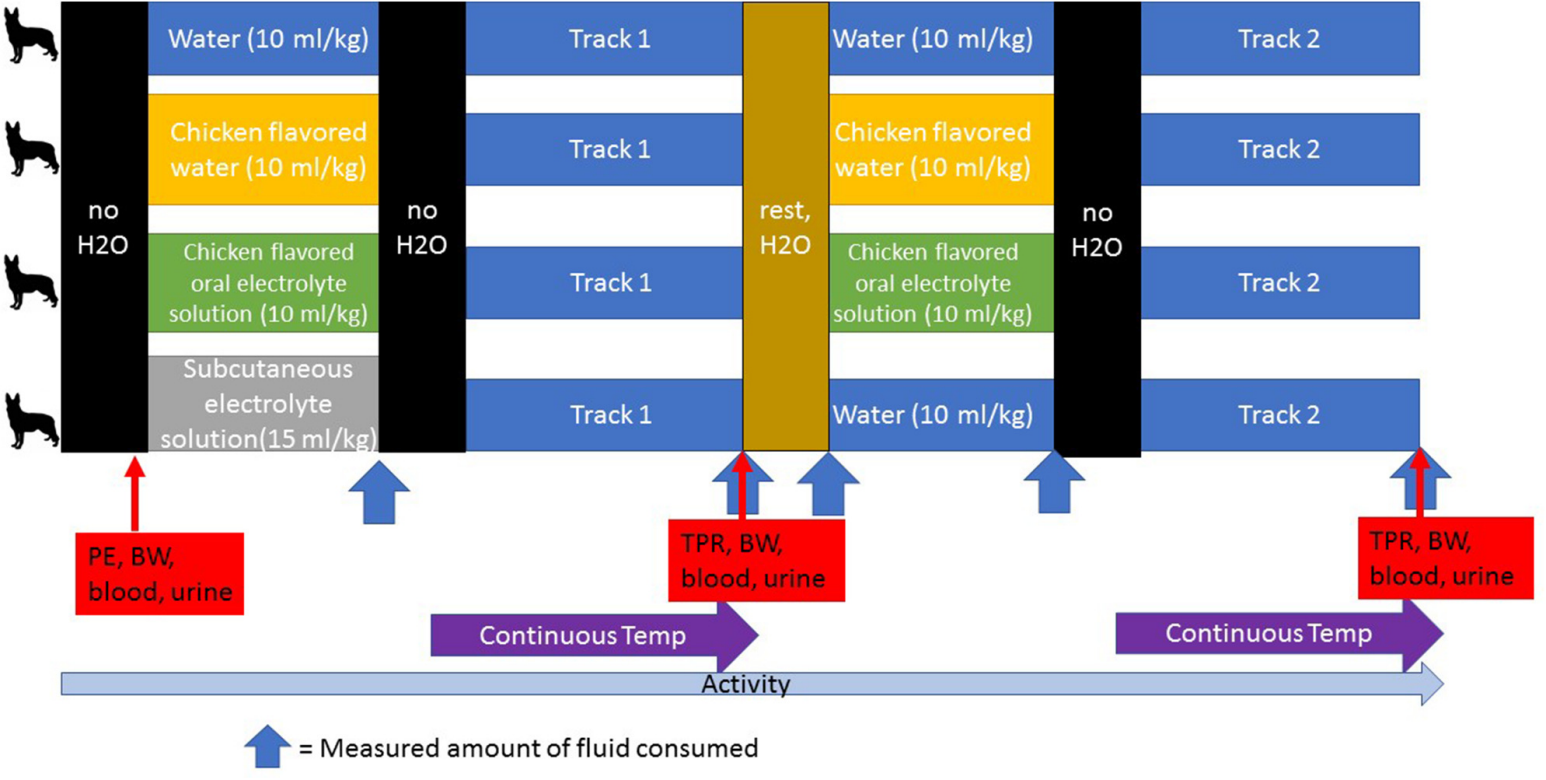
A schematic of the study design. Water was restricted at designated times (no H_2_O). An initial physical examination (PE), body weight measurement (BW) and blood and urine collection were performed at the start of each day. Dogs were randomly assigned to the treatment groups for each day and in this cross-over design, all dogs participated in each treatment group. Fluid consumption was measured after each dog was given its assigned treatment, after each track during which the handler kept track of the amount of water consumed, and during the rest period. Temperature was monitored continuously during the tracking exercise. Temperature (T), pulse (P), respiration (R), and body weight (BW) were measured and blood and urine were collected after each track. Activity was monitored continuously during each study day.

The treatment protocols were assigned randomly, and each pair of dogs had their access to water restricted either 1 h (first 2 dogs) or 2 tracks prior to their turn and received their assigned hydration strategy during the track run preceding theirs or 30 min prior to tracking (first 2 dogs) (Figure 2). The dogs were given either W, OES (Hydrolyte, Advanced Nutritional Support, Elka Park, NY, USA.), CHK (Chicken flavoring used in Hydrolyte, Advanced Nutritional Support, Elka Park, NY, USA.), or SCE (Plasmalyte A, Abbott Laboratories, North Chicago, IL, USA). The volume of assigned fluid given was 15 mL/kg for SCE and 10 mL/kg for W, OES, and CHK (See Table 1 for the composition of each fluid). After administration of the assigned fluid, water was restricted prior to launch. Each handler carried a water (H_2_O) source on the track that was measured before and after the track was finished. Handlers offered water to their dogs as they would normally while working. During tracking, the water was poured into a portable bowl and the handlers were instructed to hold the bowl and minimize any loss. Residual water was replaced into a wide mouth water bottle for measurement in a graduated cylinder at the end of the track. Water consumption was quantified during each dog's rest period until the second round of tracks began wherein the same schedule was implemented: removing access to water two tracks prior to launch and offering the same treatment protocol one track prior to launch. Dogs on the SCE protocol only received subcutaneous fluids before their first track and were offered oral water by weight (10 mL/kg) one track prior to launch of their second track.

**Table 1:** Measured and reported electrolyte composition of OES, SCE, and CHK.

**Ingredient**	**OES measured**	**SCE reported**	**CHK measured**
Sodium (mmol/L)	87	140	unmeasurable
Potassium (mmol/L)	7.4	5	2.7
Chloride (mmol/L)	67	98	unmeasurable
Buffer	Bicarbonate 8 mmol/L	Acetate 27 mEq/L Gluconate 23 mEq/L	Bicarbonate <5 mmol/L
Magnesium (mmol/L)	4.4	1.5	1.4
Glucose (mmol/L)	18	0	<0.6
Osmolality (mOsm/L)	206^*^	294	unmeasurable
Effective strong ion difference (mEq/L)^†^	27.4	47	unmeasurable

* *Osmolality was calculated as 2([Na^+^] + [K^+^]) + (glucose), where brackets represent concentration*.

† *Effective strong ion difference = [Na^+^] + [K^+^] – [Cl^−^]*.

### Data Collected

#### Dog Fluid and Food Intake

The fluid volumes offered and consumed were documented at the beginning of each track and rest cycle. The total fluid volume was recorded for each dog for each study day: fluids administered as well as water consumed for the SCE treatment, water intake for the W treatment, water plus OES for the OES treatment, and water plus chicken-flavored water for the CHK treatment. The fluids were measured using a graduated cylinder and offered in a bowl under supervision to limit any loss from spillage. Any remaining fluid was then again measured with a graduated cylinder to determine how much was consumed. Any food consumed by the dogs during the work day was recorded. Dogs were maintained on their normal feeding schedule, except for a small amount of canned dog food associated with the ingestion of the internal core temperature sensing capsules (CorTemp system, HQInc Wireless Sensing Systems & Design; Palmetto, FL, USA.).

#### Dog Physiologic Parameters

A physical examination was performed on each dog at the beginning of each day. Temperature, pulse or heart rate, and respiratory rate were obtained at the end of each track. If any dog exhibited signs of physical distress and/or was unable to maintain adequate hydration as evidenced by weakness, persistent tachycardia, poor pulse quality, and prolonged capillary refill time, it was to be removed from the study and treated appropriately.

#### Dog Activity

Each dog was assigned its own activity monitor that used omni-directional accelerometers (version 3.1, Actical®, Respironics, Koninklijke Philips Electronics, Bend, OR, USA) to collect quantitative activity data. This monitor has been validated in dogs (17) and used in a several prior exercise studies (16, 18). The activity monitors were secured to standard flat buckle collars and placed on all dogs upon arrival onsite each morning. The monitors were worn throughout the day and removed following the final track of the day.

### Environmental Parameters

Ambient temperature and percent humidity were measured every 15 min with a wireless weather station.

### Core Body Temperature

Core body temperature (gastrointestinal) was monitored using internal temperature sensing capsules. Capsules were administered in a small amount of food and, as long as the original capsule was present and transmitting appropriately, a new capsule was not administered until the old capsule passed. Temperature-recording monitors for continuous temperature readings were attached to each dog's harness just prior to launch and removed after the track ended. Intermittent readings were taken following each track as necessary.

### Body Weight, Serum Chemistry, Hematology, and Urine Measurements

The following samples were collected at the beginning of the study day and at the end of both tracking sessions: body weight, blood, and urine. Weight in kilograms was obtained using a walk-on electronic scale (Jorvet J0825PM, JorVet Walk on Scale 36“; Jorgensen Labs, Loveland, Colorado) that was calibrated twice daily. Peripheral venous blood samples from the saphenous or cephalic veins (3 mL) were anticoagulated with Li heparin for use in a point of care blood analyzer (Abaxis veterinary research laboratories, Union City, CA; CG8+ ISTAT cartridge, Abaxis, Union City, California) to measure pH, partial pressure of carbon dioxide (pCO_2_), sodium (Na), potassium (K), ionized calcium (iCa), glucose (Glu), hematocrit (Hct), and bicarbonate (HCO_3_). Lactate blood levels were analyzed using a handheld lactate meter (Lactate Scout, EKF Diagnostics, Penarth Cardiff). All remaining blood was centrifuged at 3,150 rpm for 5 min; the plasma was drawn off in two samples, placed in cryotubes and frozen on dry ice for future analysis. Blood urea nitrogen (BUN), creatinine (Creat), creatine kinase (CK), and chloride (Cl) were measured from blood samples collected at baseline (morning exam) and after completion of Track 2 at a veterinary clinical laboratory (Clinical Laboratory, M. J. Ryan Veterinary Hospital, University of Pennsylvania, Philadelphia PA) after study completion. Urine specific gravity was measured onsite with a handheld refractometer on free catch, midstream urine samples and the remaining sample was refrigerated for future analysis. Urinary sodium and urinary creatinine were measured from baseline urine collection and urine collected after completion of Track 2 samples at a veterinary clinical laboratory (Clinical Laboratory, M. J. Ryan Veterinary Hospital, University of Pennsylvania, Philadelphia PA) after study completion to calculate the fractional excretion of sodium (FeNa) at those two timepoints. Fractional excretion of sodium was calculated as described by Hinchcliff et al. (19) and used in our previous study (16).

FeNa = (UNa^*^Screat)/(SNa^*^Ucreat) ^*^100

Where UNa and SNa are the concentrations of sodium in urine and serum, respectively, and Screat and Ucreat are the concentrations of creatinine in serum and urine, respectively.

### Statistical Analyses

This study was conducted with a randomized crossover design, and the 4 interventions were assigned at random, once to each of 7 dogs; the random assignment by itself justified the permutation tests we employed, assuming “no interference” (20). For this study, no interference means that the intervention assigned at random to one dog had no effect on any other dog.

To address the within dog dependence, treatment sums of squares were computed within dogs, and the residual sum of squares had the between dog sum of squares removed. For a distribution free approach, we applied permutation ANOVA tests approximated by Monte Carlo methods (21). The very large number possible permutations effectively precluded exact tests (10,000 permutations were used for each test).

For each response variable, we evaluated all possible contrasts between our 4 interventions, searching for the greatest contrast. Beyond usual concerns about statistical tests based on multiple comparisons, “cherry picking” the greatest contrasts required statistical adjustments for post-selection statistical inference. We applied a special case of generalized maximally selected statistics that when coupled with permutation tests, provided proper statistical inference. The key requirement was that all possible contrasts were computed (22). To this end, we used the procedure COIN (23) available in the programming language R (R: a Language and Environment for Statistical Computing, R Foundation for Statistical Computing, Vienna, Austria.) to analyze the effect of hydration strategy on the following parameters: total fluid intake (sum of consumption before Track 1, during Track 1, after Track 1, before Track 2, during Track 2, and after Track 2), peak core body temperature reached over the study day, and the difference between parameters after Track 2 compared to baseline — weight, Na, Cl, K, iCa, pH, HCO_3_, pCO_2_, Glu, lactate, BUN, Creat, CK, Hct, USG, and FeNa.

Descriptive data were reported as median and range. To determine if there was a significant difference in absolute (rather than change) blood or urine parameters between baseline and Track 2, a two-way repeated measures ANOVA or Wilcoxon Signed Rank Test was run on values for the following parameters: weight, peak core body temperature, BUN, CK, FeNa, and USG.

To address whether the environmental conditions affected peak body temperature, we plotted the peak core body temperature measured for Track 1 and Track 2 against both ambient humidity and ambient temperature and analyzed the regression coefficient.

## Results

Four intact males and 3 spayed females participated in this study. Two of the females were Belgian Malinois, 2 of the males were German Shepherds, one female and one male were Labrador Retrievers, and one male was a Belgian Malinois-German Shepherd cross. All dogs were between 2 and 7 years old: median age 6 years. The dogs' diets were one of 2 commercial dry diets (Science Diet Active Adult or Science Diet Advanced Fitness, Hill's Pet Nutrition, Topeka, KS, USA). All dogs were assessed as a 5 or 5.5 on the 1–9 body condition scoring system (24). All dogs except one who arrived from Harlingen, Texas were located in El Paso, Texas prior to the start of the study. As a specialized unit, the BORSTAR dogs do not work on a routine schedule. To account for differences in acclimation to activity, we documented the last time the dog had been deployed to track or perform any other duty related work. At the start of the study it had been a mean of 3.5 ± 2.3 weeks since the dogs last worked. All dogs appeared normal on physical examination at the start of each study day, and no dogs demonstrated signs of physical distress or illness that prevented them from participating for the full study day. Dogs received no food while working each study day except for that associated with placement of the internal temperature sensing capsules. Throughout the entire duration of the study, handlers reported 1 case of diarrhea (W), 2 cases of sore paws (two different dogs on OES), 3 episodes of decreased appetite all in the same dog (W, OES, SCE), and 1 case of decreased urination (CHK).

Total activity counts for each dog for each track were divided by the minutes spent tracking, for Track 1 activity counts ranged from 1,390–4,198 (median 2,386) counts per minute. For Track 2, activity ranged from 1,217–4,641 (median 2,373) counts per minute. Average track time for Track 1 was 24.3 min and ranged from 12–38 min. Average time for Track 2 was 25.2 min and ranged from 8–72 min.

Median baseline core body temperature across all study days was within normal limits but Track 1 and Track 2 peak temperatures were significantly elevated from baseline (Table 2) (*p* < 0.05). Peak core body temperatures while tracking ranged from 39.95 to−42.88°C (103.91–109.18°F). During the study days, ambient temperature and humidity ranged from 24.7–30.6°C (76.5–87.0°F) and 37.0–63.3%, respectively for Track 1. For Track 2, ambient temperature and humidity ranged from 28.3–37.1°C (83.0–98.8°F) and 23.3–60.0%; see Table 2 for mean environmental parameters and baseline and treatment and track specific examination parameters. Accurate respiratory rates were not obtained due to the dogs' panting.

**Table 2:** Median core body temperature, pulse rate, and weight of all dogs over all study days as a function of time and treatment group.

	**Baseline**	**Track1**					**Track2**				
		**Overall, *N* = 28**	**W, *N* = 7**	**OES, *N* = 7**	**CHK, *N* = 7**	**SCE, *N* = 7**	**Overall, *N* = 28**	**W, *N* = 7**	**OES, *N* = 7**	**CHK, *N* = 7**	**SCE, *N* = 7**
Weight (kg)	31.8 (30.4–33.7)	32.1 (30.4, 33.4)	31.7 (30.3, 32.8)	32.0 (30.4, 33.2)	32.5 (30.6, 33.9)	32.6 (30.6, 33.4)	31.7 (30.0, 32.7)^*^	31.4 (29.6, 32.2)	32.0 (29.9, 32.6)	32.0 (30.6, 33.5)	30.6 (30.2, 33.1)
Pulse (bpm)	102 (96–120)	140 (120, 150)	130 (120, 140)	140 (118, 148)	150 (130, 175)	140 (119, 144)	140 (120, 150)	140 (122, 142)	142 (131, 154)	150 (145, 158)	120 (110, 142)
Peak core temperature (°C)	38.47 (38.12–38.83)	41.15 (40.77, 41.73)^*^	40.96 (40.61, 41.20)	41.22 (40.80, 41.67)	41.96 (41.37, 42.37)	40.79 (40.69, 41.23)	41.30 (40.87, 41.86)^*^	41.59 (41.02, 42.36)	41.16 (40.96, 41.82)	41.57 (40.77, 41.81)	41.30 (41.10, 41.44)
Peak core temperature (°F)	101.25 (100.62–101.90)	106.07 (105.39, 107.12)^*^	105.72 (105.10, 106.16)	106.20 (105.43, 107.00)	107.52 (106.47, 108.26)	105.42 (105.25, 106.22)	106.34 (105.56, 107.34)^*^	106.86 (105.83, 108.25)	106.08 (105.72, 107.28)	106.82 (105.38, 107.26)	106.34 (105.98, 106.59)
Track time (min)		24.0 (19.5, 29.0)	27.0 (18.5, 27.0)	22.0 (19.2, 29.2)	27.0 (21.0; 29.5)	21.0 (18.5; 29.2)	22.5 (13.5, 31.5)	20.0 (15.0, 27.8)	25.0 (19.5; 32.5)	25.0 (16.8, 41.0)	12.0 (12.0; 22.5)
Ambient temperature°C (°F)		28.0 ± 1.6 (82.5 ± 2.8°F)					32.4 ± 2.2 (90.3 ± 4.0°F)				
Humidity		50.2 ± 7.7 %					38.9 ± 8.8%				

*Core body temperature for tracks are presented as the median peak value reached within each tracking session for each treatment group. Pulse rate and body weight were measured at baseline and following completion of each track. Statistics, Median (IQR) Mean +/- SD (Ambient Temperature and Humidity); W, water; OES, chicken-flavored oral electrolyte solution; CHK, chicken-flavored water; SCE, subcutaneous electrolyte solution*;

* *, significantly different from baseline*.

### Effect of Hydration Strategy on Fluid Intake

Total fluid intake was influenced by hydration strategy (*p* = 0.0208) when controlling for individual dog. Contrast analysis revealed that fluid intake for the W treatment, which is considered the baseline fluid consumption, was significantly different from intake with the other three strategies. Mean total fluid intake by hydration protocol was 49.1 (41.1, 50.5) mL/kg for W, 59.7 (52.4, 74.4) mL/kg for CHK, 62.9 (56.2, 74.4) mL/kg for SCE, and 83.3 (56.8, 85.6) mL/kg for OES. All dogs drank the full pre-tracking fluid volume for both Track 1 and Track 2 when they were offered OES. When offered CHK, all dogs drank the full pre-hydration amount for Track 2, and all but one dog drank the full pre-hydration amount for Track 1. Additional Na load for OES was 1.74 mEq/kg and 2.1 mEq/kg for SCE. No measurable amounts of Na were provided by W or CHK.

### Influence of Hydration Strategy on Peak Temperature and Weight Loss

The dogs' median peak temperature from both tracks and each treatment group are reported in Table 2. Although we were unable to detect an overall effect of hydration strategy on peak temperature (*p* = 0.1316), when we compared the means of electrolyte enriched (i.e., OES and SCE) vs. electrolyte free (i.e., CHK and W) solutions to address whether the presence of electrolytes influenced peak core body temperature, the *p* value was not significant at 0.07. When controlling for dog and hydration strategy, there was no significant difference in the dogs' peak temperature for Track 1 vs. Track 2. We were not able to detect an effect of hydration strategy on the change in weight when comparing baseline values to values post Track 2.

### Effect of Hydration Strategy on Blood and Urine Parameters

Hydration strategy had a significant impact on the change in CK (*p* = 0.0361) with a significant contrast between CHK and other hydration strategies (Table 3) (*p* = 0.0361). We were not able to detect an effect of hydration strategy on change in Na, Cl, K, iCa, pH, pCO_2_, Glu, lactate, BUN, Creat, Hct, USG, and FeNa. When we compared the average change in HCO_3_ between the hydration strategies, the *p* value was not significant at *p* = 0.0652 with contrast between W and the other three hydration strategies. See Table 3 for blood chemistry, hematology, and urinary parameters at baseline and after each tracking session for each hydration strategy.

**Table 3:** Blood chemistry, hematology, and urinary parameters of all dogs at baseline and after each tracking session.

**Characteristic**	**Track 1**					**Track 2**				
	**Overall, *N* = 28**	**W, *N* = 7*^1^***	**OES, *N* = 7*^1^***	**CHK, *N* = 7*^1^***	**SCE, *N* = 7*^1^***	**Overall, *N* = 28**	**W, *N* = 7*^1^***	**OES, *N* = 7*^1^***	**CHK, *N* = 7*^1^***	**SCE, *N* = 7*^1^***
Sodium (mmol/L) *RR 139–150***B: 147 (145, 148)**	147 (146, 148)	146 (145, 148)	148 (146, 150)	146 (146, 147)	148 (148, 148)	144 (144, 146)	144 (144, 146)	145 (144, 148)	144 (144, 147)	144 (144, 146)
Potassium (mmol/L)*RR 3.4–4.9***B: 4.1 (4.0, 4.3)**	3.85 (3.68, 4.10)	3.80 (3.75, 4.00)	3.80 (3.65, 3.85)	4.00 (3.75, 4.10)	4.10 (3.60, 4.15)	3.60 (3.48, 3.80)	3.70 (3.60, 3.95)	3.50 (3.40, 3.75)	3.50 (3.40, 3.60)	3.80 (3.65, 3.85)
Ionized Calcium (mmol/L)*RR 1.12–1.40***B: 1.33 (1.32, 1.34)**	1.23 (1.21, 1.26)	1.27 (1.23, 1.27)	1.22 (1.17, 1.25)	1.22 (1.17, 1.25)	1.23 (1.21, 1.25)	1.19 (1.15, 1.22)	1.20 (1.16, 1.23)	1.16 (1.15, 1.21)	1.17 (1.13, 1.20)	1.21 (1.18, 1.24)
pH*RR 7.35–7.45***B: 7.41 (7.38, 7.44)**	7.53 (7.51, 7.59)	7.52 (7.48, 7.56)	7.56 (7.52, 7.59)	7.54 (7.53, 7.61)	7.56 (7.47, 7.57)	7.63 (7.58, 7.70)	7.61 (7.53, 7.72)	7.63 (7.59, 7.69)	7.65 (7.59, 7.72)	7.61 (7.59, 7.65)
Bicarbonate (mmol/L)*RR 15–23***B: 19 (18, 20)**	13.25 (11.43, 15.33)	14.80 (13.10, 15.15)	11.70 (11.15, 14.60)	11.20 (10.00, 13.85)	15.30 (12.80, 15.65)	11.60 (10.50, 12.20)	10.70 (10.55, 11.70)	11.20 (10.45, 11.80)	12.10 (11.80, 12.25)	11.50 (10.45, 12.30)
Partial pressure of CO_2_ (mmHg)*RR 35-38***B: 31 (28, 33)**	14.9 (12.3, 20.1)	17.2 (13.6, 20.5)	13.1 (11.4, 17.9)	13.3 (9.7, 16.8)	16.5 (14.1, 21.6)	10.7 (9.3, 12.4)	10.5 (8.9, 13.3)	10.0 (8.9, 11.2)	10.2 (9.6, 12.1)	11.0 (10.1, 12.6)
Glucose (mmol/L)*RR 3.3–6.4***B: 5.0 (4.7, 5.5)**	5.2 (4.8, 5.7)	5.1 (4.8, 5.5)	5.2 (4.7, 5.9)	5.1 (4.6, 5.8)	5.2 (5.0, 5.5)	4.9 (4.6, 5.4)	5.4 (4.5, 5.5)	4.9 (4.6, 4.9)	5.0 (4.5, 5.3)	4.9 (4.8, 5.5)
Lactate (mmol/L)*RR <2.0***B: 1.4 (1.0, 1.6)**	2.65 (1.90, 3.32)	2.50 (2.15, 3.05)	2.60 (1.65, 3.80)	4.40 (2.80, 4.55)	2.30 (1.85, 2.90)	2.95 (2.48, 3.70)	3.00 (2.80, 4.00)	2.70 (2.30, 3.30)	3.40 (1.80, 3.75)	3.00 (2.50, 3.20)
Hematocrit (%)*RR 35–50***B: 47 (44, 49)**	47 (46, 50)	47 (45, 47)	47 (47, 49)	48 (48, 51)	47 (46, 50)	46 (43, 47)	46 (44, 48)	45 (43, 46)	46 (46, 50)	43 (43, 46)
Urine Specific Gravity**B: 1.065 (1.046, 1.071)**	1.055 (1.042, 1.068)	1.057 (1.046, 1.066)	1.055 (1.039, 1.069)	1.050 (1.022, 1.063)	1.054 (1.048, 1.069)	1.024^*^ (1.014, 1.046)	1.041 (1.014, 1.045)	1.032 (1.018, 1.047)	1.016 (1.014, 1.017)	1.045 (1.018, 1.062)
Chloride (mmol/L)*RR 109–120***B: 118 (115, 119)**						119 (117, 121)	120 (118, 120)	119 (117, 121)	118 (116, 122)	118 (117, 122)
Blood Urea Nitrogen(mmol/L)*RR 3.6–9.3***B: 6.4 (5.7, 7.1)**						6.4 (5.7, 7.1)	6.8 (5.7, 6.8)	6.4 (5.3, 7.1)	6.8 (6.4, 7.1)	6.1 (5.7, 7.1)
Creatinine (umol/L)*RR 62–159***B: 115 (97, 115)**						114.9 (113.1, 123.8)	123.8 (114.9, 123.8)	114.9 (106.1, 123.8)	123.8 (114.9, 132.6)	114.9 (110.5, 119.3)
Creatine Kinase (U/L)*RR 46–467***B: 66 (50, 88)**						158^*^ (128, 246)	124 (114, 217)	172 (147, 302)	322^a^ (152, 562)	150 (136, 183)
Fractional Excretion of Sodium (%)**B: 0.26 (0.11, 0.37)**						0.70^*^ (0.39, 1.28)	0.67 (0.28, 1.11)	1.04 (0.53, 1.51)	0.69 (0.40, 0.96)	0.67 (0.45, 1.15)

1 *Statistics presented: median (IQR) W, water; OES, chicken-flavored oral electrolyte solution; CHK, chicken-flavored water; SCE, subcutaneous electrolyte solution; RR, reference range; B, baseline (pretracking value); a, significantly different from the other treatment strategies (p = 0.0361)*;

* *, significantly different from baseline*.

*All values are reported in international units Reference range provided for point of care blood analyzer cartridge, (36) (CG8+ ISTAT cartridge, Abaxis, Union City, California; Na, K, iCa, pH, HCO3, pCO2, Glu, Hct) and veterinary clinical laboratory (Clinical Laboratory, M. J. Ryan Veterinary Hospital, University of Pennsylvania, Philadelphia PA, USA; Cl, BUN, Creat, CK)*.

### Effect of Activity and Environmental Conditions

Median body weight was significantly lower after Track 2 compared to baseline when controlling for hydration strategy (*p* = 0.041). USG, FeNa, and CK also varied significantly between baseline and Track 2 (*p* = 0.016, *p* = 0.005, *p* < 0.001, respectively). We were unable to detect a difference in BUN after Track 2 compared to baseline BUN. Peak core body temperature was not associated with either ambient humidity or ambient temperature.

## Discussion

The dogs in this study faced harsher conditions and exhibited more rigorous activity than dogs in a previous study (16). They tracked in an open arid environment with no access to shade and displayed higher heart or pulse rates, higher body temperatures, and higher activity counts. The highest core body temperature recorded in the previous study was 40.3°C (104.5°F) (16) while the dogs in the current study had average peak temperatures above 41.1°C (106°F). Accelerometer data has been categorized into sedentary, walking, and trotting activity in pet dogs, although the cutoff between trotting and more rigorous activity was not defined (17). The average speed of tracking was 2.4 miles/hr with activity counts for most of the tracking sessions above the 1,751 counts per minute threshold indicative of trotting (17). In comparison, the dogs in the previously published hydration study had a mean activity of 750 counts/min although this did include rest intervals, but they were rarely observed trotting (16). Based on activity counts and visual observation, the tracking activity of the dogs in this study could be classified as moderate intensity.

In this study of dogs undergoing moderate activity while tracking in hot and arid conditions at the border in El Paso, Texas, the 4 hydration strategies had negligible effects on blood and urinary parameters. Of all the hematological, blood serological, and urinary parameters measured, hydration strategy only had a statistically significant impact on the change in CK. The increase in CK in the dogs given chicken-flavored water, suggests that this strategy is associated with a higher degree of muscle injury. Small, but significant changes in CK were also reported after a 4-h search and rescue exercise (Spoo, 2015). The post track CK for all but 2 dogs in the study was within the normal reference range and no dog showed signs of rhabdomyolysis. Bruchim et al. (2019) reported that military working dogs with a history of heat stroke, had higher CK after indoor (but not outdoor) exercise compared to military working dogs without a history of heat stroke. None of the dog handlers in the current study reported a history of heat injury or heat stroke, and since it was a cross over design, any individual dog effects are accounted for. Although the mechanism of muscle injury cannot be determined in this study, hydration strategy did have a significant impact on total fluid intake. The higher fluid consumption without electrolytes in the CHK strategy may be a contributing factor.

Dogs consumed less fluids with the W treatment suggesting the benefit of pre-treatment with an alternate hydration strategy. Dogs on the SCE treatment were guaranteed to receive 15 mL/kg of fluids prior to the start of Track 1 while dogs voluntarily consumed all the pre-tracking fluids before both sessions when offered OES or CHK in all but one case. While flavoring likely accounted for the willingness to consume CHK and OES pre-tracking fluid, the added sodium and chloride in the OES and SCE fluids likely drove subsequent water intake as studies have demonstrated that dogs will increase their water consumption following increased salt intake (25–27).

Serum Na did not vary by hydration strategy so despite the additional Na load from the OES and SCE protocols, dogs were able to maintain normal serum Na levels. The FeNa did not vary by hydration strategy and was increased after Track 2 compared to baseline regardless of hydration strategy. Similarly, USG did not vary by hydration strategy but was lower after Track 2 compared to baseline. This finding was in contrast to the expectation that the USG would increase to conserve water while dogs were exercising in a hot environment. The decrease in USG following repeated tracking exercises suggests that the dogs were either more hydrated or that there was a cumulative effect of exercise on the dogs' ability to concentrate their urine. The average weight after Track 2 was lower than baseline which suggests that the dogs were losing water not becoming more hydrated. Both the decrease in USG and the increase in FeNa likely represent a cumulative effect of exercise. We have previously documented an increase in FeNa in Border Patrol dogs following mild to moderate exercise (16). Increased excretion of Na may contribute to increased water loss due to osmotic drag which would cause a subsequent decrease in USG, a loss in weight, and potentially a decrease in serum Na.

It has been suggested that electrolyte supplementation is unwarranted for working dogs given that they pant rather than sweat to cool themselves (28). However, this study found some potential benefit and no electrolyte abnormalities associated with electrolyte supplementation (OES or SCE) and is consistent with previous study (16) which found no adverse effects associated with either oral electrolytes or subcutaneous fluids. In a field study of racing sled dogs by Hinchcliff et al. (19), prolonged exercise did lead to hyponatremia. In contrast to our dogs, the sled dogs had decreased urinary sodium and no change in urine osmolality. It is possible that the combination of prior fitness training, unique diet, endurance activity and cold temperatures may elicit a different physiologic response. We did not measure aldosterone or vasopressin; therefore, we cannot directly compare the results. During exercise, sodium can also be lost through salivation (29) and urine. The prior studies of Hinchcliff et al. (19) and Otto et al. (16) suggest that electrolyte supplementation may have value in exercising dogs. The results of this study and Otto et al. (16) suggests electrolyte supplementation is not only safe but may also be beneficial by providing sodium to counter endogenous losses and helping to drive consumption of fluids necessary to enhance evaporative cooling.

We did not detect a correlation between ambient temperature or ambient humidity and peak core body temperature which is consistent with reports from a previous study in working dogs (18). The dogs were all from warm climates so the independence of core body temperature from ambient conditions may reflect acclimation to the environment and work. The dogs' average peak core body temperatures during tracking were above 41°C (105.8°F) which is conventionally considered indicative of heat stroke and potential for permanent brain damage (30–32) although this temperature-based criterion has been criticized (33). Despite the high core body temperatures recorded throughout this study, only one dog showed signs of heat stress. On the first study day, one dog reached a core body temperature above 42.8°C (109°F) and displayed unsteadiness and shade-seeking behavior upon completion of Track 1. He was wet down and closely monitored but recovered uneventfully and was able to complete the second tracking session that day although his Track 2 time was slow (over an hour to complete the one-mile track). Later analysis of his blood CK level (3,391 U/L) suggested muscle injury, although there was no evidence of pigmenturia. Another dog that same day reached an even higher temperature of 42.88°C (109.18°F), yet did not have any behavioral or blood value changes indicative of heat stress. Similarly, during many of the other tracking sessions throughout the study, dogs reached core body temperatures above 41°C (105.8°F) with no signs of heat stress. Other studies on working dogs have also reported core body temperatures (measured with internal core temperature sensing capsules) above 41.1°C (106°F) with no clinical signs of heat stress (18, 34). Heat-acclimation has been shown to elevate the core body temperature threshold for thermal injury in rats (35), and increased transcription of heat shock proteins has been documented in MWD following acclimatization and physical training (10). Likely, heat-acclimation, acclimatization, and exercise conditioning accounts for the dramatically elevated temperatures observed in working dogs without subsequent heat stroke, but further studies are needed to sort out the role and underlying mechanisms of these factors in regard to this phenomenon.

This study looked at various hydration methods strictly as pre-treatments, and only water was offered to the dogs while they tracked. Some SAR dog handlers report administering SCE prophylactically prior to a shift (12), but repeated SCE administration is impractical. Additionally, OES and CHK can spoil in the heat after preparation. Some individuals flavor water to increase palatability; however, the electrolyte content can be highly variable, and, as seen in the dogs in the CHK strategy, the absence of electrolytes may be associated with an increased risk of muscle injury.

There are several limitations to this study. Weight loss was used as a proxy for water loss through evaporation, urine, or saliva. However, these losses were not directly measured so weight loss could potentially have been due to other factors (e.g., defecation or scale fluctuations). The amount of water consumed may have been impacted by loss during drinking; however, since the study was a crossover design, dogs that were more likely to splash water would have done so in each treatment arm. This study relied on a small number of dogs that were all from Texas, and represented different ages and breeds. Our findings may not apply to dogs from cool climates. The variability of ages and breeds are representative of the working dog population, but the variability may have masked some outcomes that could have been significant in a more uniform population. We were not able to address carry over effects since we only analyzed changes that occurred over study days. It is possible that the small sample size limited our ability to detect a significant impact of electrolyte-enriched fluids on peak core body temperature. Our data suggests that pre-treatment with electrolyte-enriched fluids may contribute to lower peak core body temperatures, but further investigation is needed, especially given the number of other factors that could contribute to peak working temperature. Finally, this study analyzed only one OES formulation, and results cannot be extrapolated to other oral electrolyte formulations as composition could drastically alter palatability, safety, and efficacy. A previous study utilizing a different OES found no increase in fluid consumption, and some dogs even refused to drink the electrolyte solution (15). More studies are needed to address the long-term effects of OES and SCE hydration protocols.

In conclusion, while this study found that hydration strategy had limited effects on blood and urinary parameters, all three alternate hydration strategies (CHK, OES, SCE) increased total fluid intake compared to W. To achieve the goal of increasing fluid intake, without the associated risk of increased muscle injury, OES and SCE pre-treatment should be considered based on field conditions and availability. Furthermore, electrolyte-enriched hydration pre-treatments (OES and SCE) may additionally help dogs to maintain a lower peak temperature although further studies are required to explore this trend.

## Data Availability Statement

The datasets generated for this study are available on request to the corresponding author.

## Ethics Statement

The animal protocols used in this study were reviewed and approved by both the University of Pennsylvania and US Army Medical Research and Materiel Command Institutional Animal Care and Use Committees (USAMRCM #SO120002, UPenn IACUC protocol #804293). Written informed consent was obtained from the owners for the participation of their animals in this study.

## Author's Note

A. COIN is a software library in R. Conditional inference procedures for the general independence problem including two-sample, K-sample (non-parametric ANOVA), correlation, censored, ordered and multivariate problems.

## Author Contributions

GN led the manuscript preparation and review. EH and RB participated in data analysis and manuscript review. LB, TD, JN, and KS participated in data collection, and manuscript review. KK participated in study design, data collection, and manuscript review. CO oversaw the design of the study, assisted with data collection, data analysis and manuscript preparation and review.

### Conflict of Interest

EH was employed by the company Dog Genetics, LLC. The remaining authors declare that the research was conducted in the absence of any commercial or financial relationships that could be construed as a potential conflict of interest.

## Abbreviations

CHK: chicken-flavored water
MWD: Military Working Dog
OES: chicken-flavored oral electrolyte solution
SAR: search and rescue
SCE: subcutaneous electrolyte solution
W: oral water.
